# Supplementary material for: Oil immersed lossless total analysis system for integrated RNA extraction and detection of SARS-CoV-2
Source: Nat Commun. 2021 Jul 14;12:4317. doi: 10.1038/s41467-021-24463-4 (PMC8280165; doi:10.1038/s41467-021-24463-4)
Supplement: Supplementary file 1 — Supplementary Information [file 41467_2021_24463_MOESM1_ESM.pdf]

Supplementary Information

Oil Immersed Lossless Total Analysis System for Integrated RNA Extraction and Detection of SARS-CoV-2

Duane S. Juang[1†], Terry D. Juang[1†], Dawn M. Dudley[2], Christina M. Newman[2], Molly A. Accola[3], William M. Rehrauer[2,3], Thomas C. Friedrich[4,5], David H. O'Connor[2,4] & David J. Beebe[1,2*]

[1]Department of Biomedical Engineering, University of Wisconsin-Madison, Madison, WI, USA

[2]Department of Pathology and Laboratory Medicine, University of Wisconsin-Madison, Madison, WI, USA

[3]UW Health Clinical Laboratories, University of Wisconsin Hospital and Clinics, Madison, WI, USA

[4]Wisconsin National Primate Research Center, University of Wisconsin-Madison, Madison, WI, USA

[5]Department of Pathobiological Sciences, University of Wisconsin-Madison, Madison, WI, USA

*Corresponding author: djbeebe@wisc.edu.

[†]These authors contributed equally to this work

Table of contents:

Supplementary Fig. 1: Graphical operation protocol for SARS-CoV-2 testing using OIL-TAS....... S-2

Supplementary Fig. 2: Extraction carryover of OIL-TAS S-2

Supplementary Fig. 3: Droplet stability of OIL-TAS S-3

Supplementary Fig. 4: Performance of OIL-TAS using a reagent freshly loaded device compared to a reagent pre-loaded device that was frozen for a week at -20 ºC S-3

Supplementary Fig. 5: Extraction equivalency of the dual output OIL-TAS device S-4

Supplementary Table 1: Clinical sample results........ S-5

Supplementary Table 2: LAMP primer sequences S-9

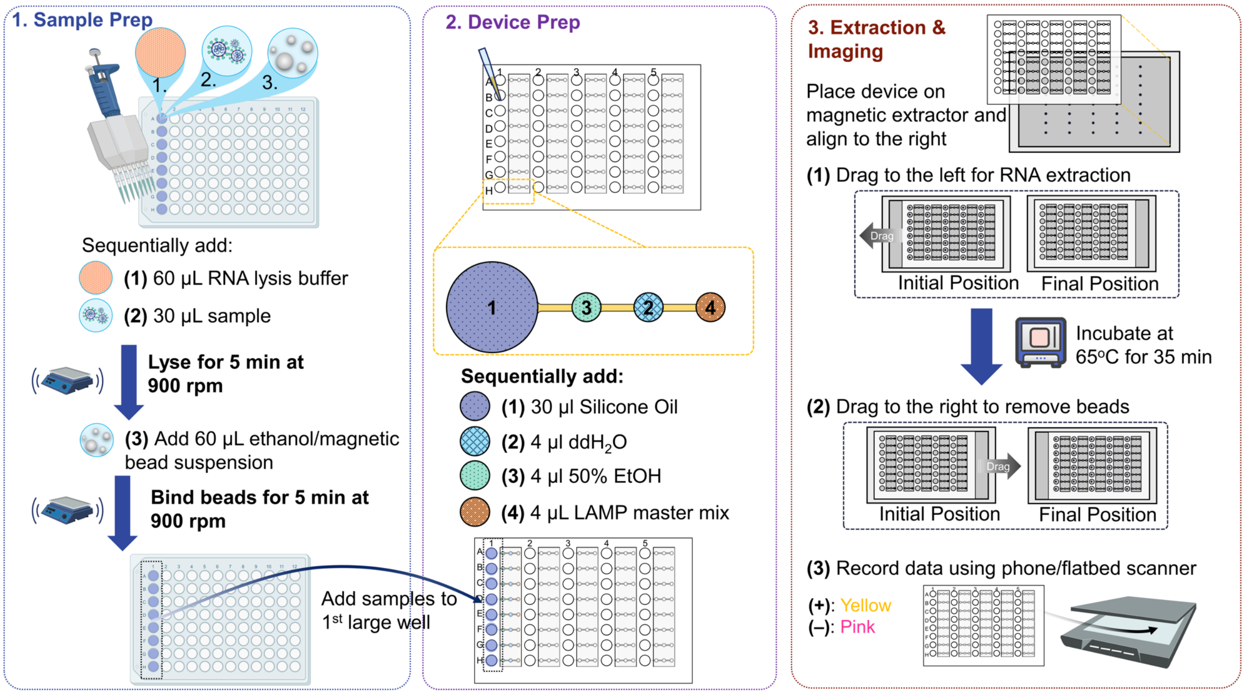

Supplementary Fig. 1 Graphical operation protocol for SARS-CoV-2 testing using OIL-TAS.

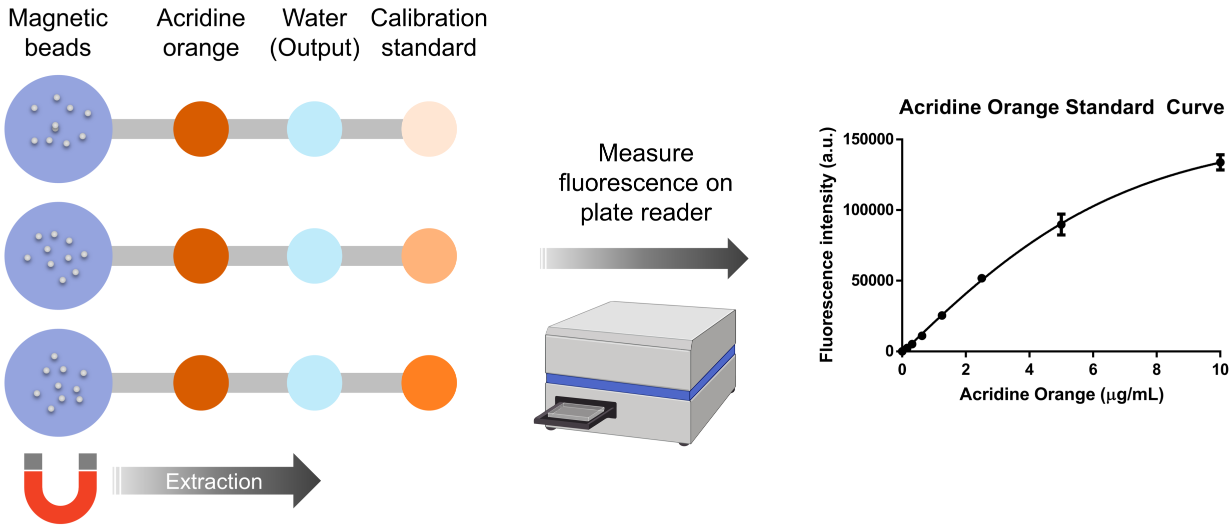

Supplementary Fig. 2 Extraction carryover of OIL-TAS. The amount of carryover was calculated by fitting the fluorescence intensity of the water (output) droplet to the serially diluted acridine orange standard curve. Source data are provided as a Source Data file.

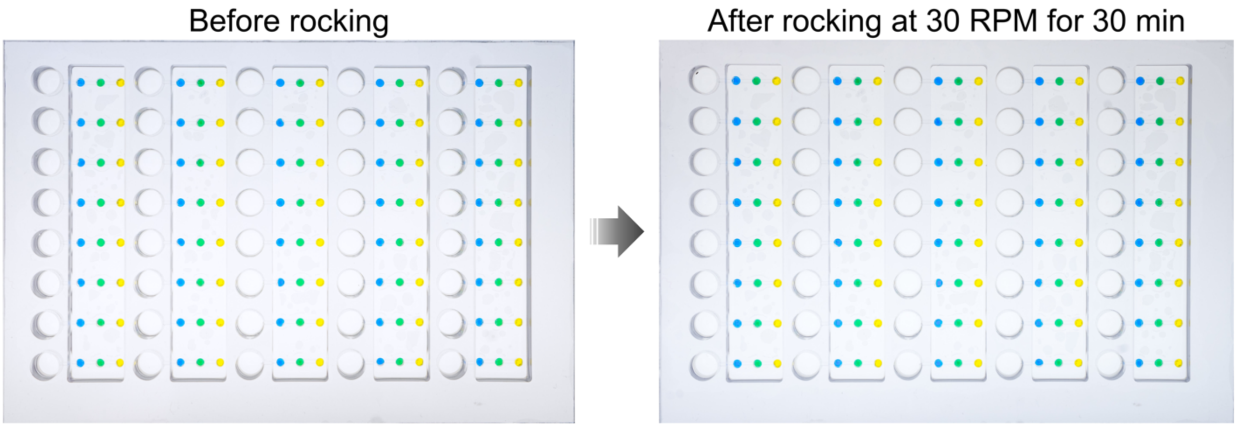

Supplementary Fig. 3 Droplet stability of OIL-TAS. No droplets were dislodged from the wells in the device after rocking on a rocking platform shaker at 30 RPM for 30 min.

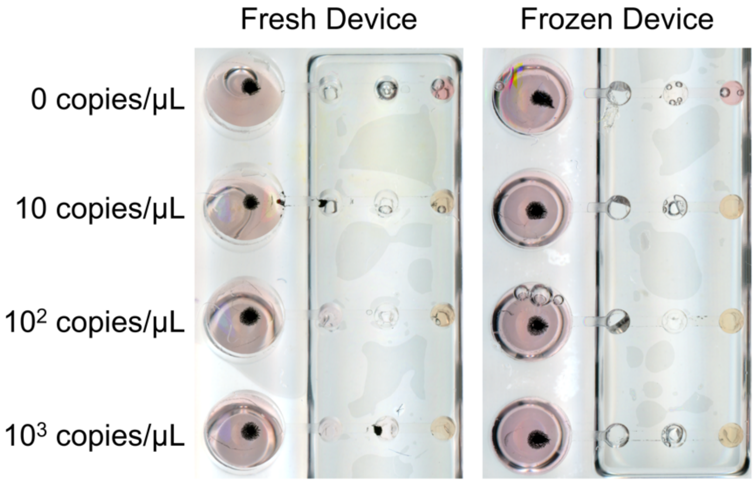

Supplementary Fig. 4 Performance of OIL-TAS using a reagent freshly loaded device (left) compared to a reagent pre-loaded device that was frozen for a week at -20 ºC (right).

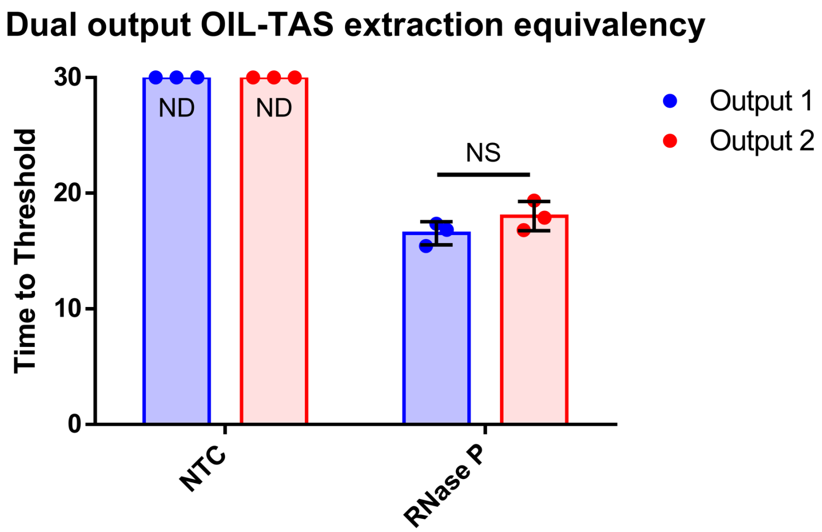

Supplementary Fig. 5 Extraction equivalency of the dual output OIL-TAS device. A sample containing RNase P RNA was extracted in parallel into detection well 1 and detection well 2 of the dual output OIL-TAS device. Extraction equivalency of the two wells was evaluated by time to threshold values of LAMP RNase P amplification. NTC: no template control, ND: Not detected, NS: Not significant, determined using 2-tailed Student's t-test. Data are represented as mean values +/- SD from 3 replicates. Source data are provided as a Source Data file.

Supplementary Table 1: Clinical sample results
(+) OIL-TAS positive, (−) OIL-TAS negative, (Δ) OIL-TAS inconclusive.
Discrepant OIL-TAS results compared with RT-qPCR are highlighted in orange.

Sample ID	Medium	RT-qPCR Ct	OIL-TAS rep 1	OIL-TAS rep 2	OIL-TAS rep 3	OIL-TAS result (n=3)
1	PBS	19.32	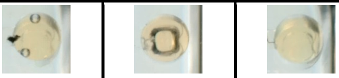			+ 3/3
2	PBS	16.53	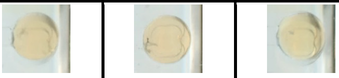			+ 3/3
3	RT M4	Neg	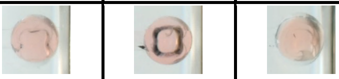			− 0/3
4	UTM	Neg	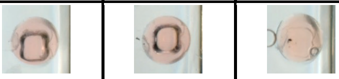			− 0/3
5	UTM	30.16	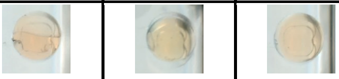			+ 3/3
6	PBS	18.71	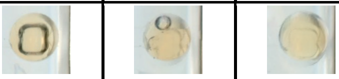			+ 3/3
7	UTM	Neg	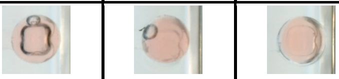			− 0/3
8	PBS	23.79	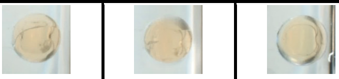			+ 3/3
9	RT M4	Neg	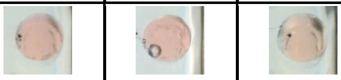			− 0/3
10	UTM	Neg	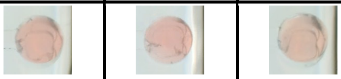			− 0/3
11	UTM	Neg	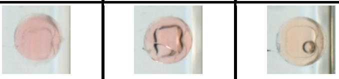			− 0/3
12	UTM	Neg	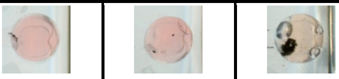			− 0/3
13	PBS	24.31	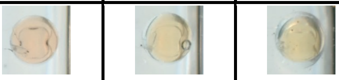			+ 3/3
14	RT M4	Neg	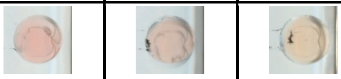			− 0/3
15	UTM	34.22	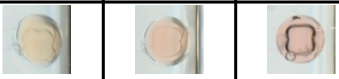			Δ 1/3
16	PBS	Neg	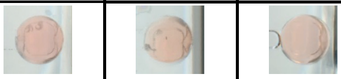			− 0/3
17	UTM	Neg	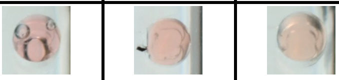			− 0/3
18	UTM	23.5	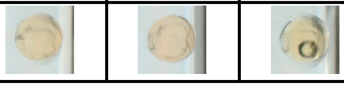			+ 3/3

19	UTM	28				+ 3/3
20	UTM	16.51				+ 3/3
21	UTM	17.3				+ 3/3
22	PBS	15.7				+ 3/3
23	UTM	39				− 0/3
24	UTM	28.9				+ 3/3
25	UTM	32				+ 3/3
26	UTM	17.5				+ 3/3
27	UTM	28.4				+ 3/3
28	UTM	23.1				+ 3/3
29	UTM	29.6				+ 3/3
30	UTM	25.8				+ 3/3
31	UTM	16.4				+ 3/3
32	UTM	19.4				+ 3/3
33	UTM	21.4				+ 3/3
34	UTM	23.2				+ 3/3
35	UTM	17.7				+ 3/3
36	UTM	20.5				+ 3/3
37	UTM	20				+ 3/3
38	UTM	21.3				+ 3/3

39	PBS	24	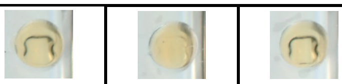			+<br>3/3
40	UTM	18.5	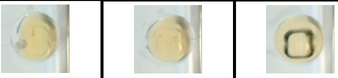			+<br>3/3
41	PBS	28.2	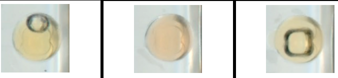			+<br>3/3
42	PBS	15.5	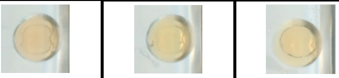			+<br>3/3
43	PBS	16.6	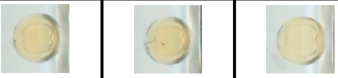			+<br>3/3
44	PBS	16.5	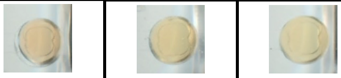			+<br>3/3
45	PBS	16.5	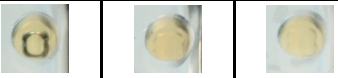			+<br>3/3
46	PBS	28.5	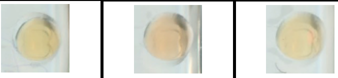			+<br>3/3
47	UTM	32.7	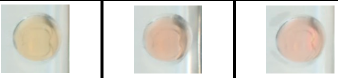			Δ<br>1/3
48	UTM	21.5	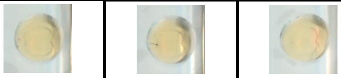			+<br>3/3
49	UTM	23.9	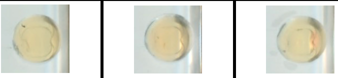			+<br>3/3
50	UTM	17.7	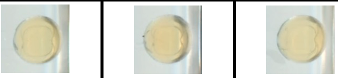			+<br>3/3
51	UTM	25.5	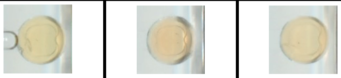			+<br>3/3
52	UTM	26.2	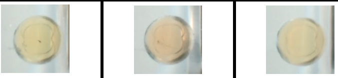			+<br>3/3
53	UTM	21.5	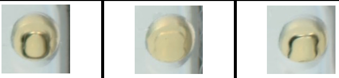			+<br>3/3
54	UTM	28.2	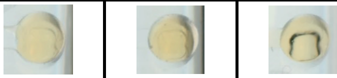			+<br>3/3
55	UTM	18.2	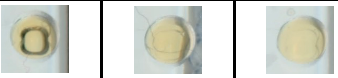			+<br>3/3
56	UTM	15.5	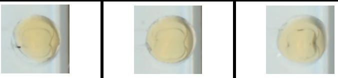			+<br>3/3
57	UTM	25.4	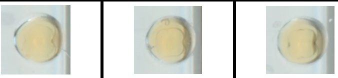			+<br>3/3
58	UTM	14.4	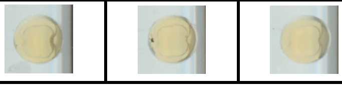			+<br>3/3

59	UTM	23.9	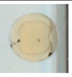	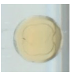	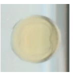	+<br>3/3
60	UTM	19.6	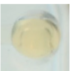	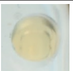	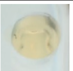	+<br>3/3
61	UTM	18.5	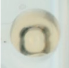	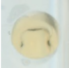	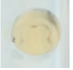	+<br>3/3
62	UTM	21.9	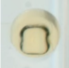	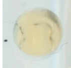	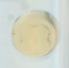	+<br>3/3
63	UTM	30.3	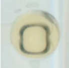	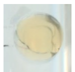	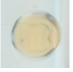	+<br>3/3
64	UTM	35.6	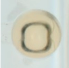	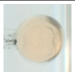	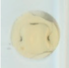	+<br>3/3
65	UTM	18.7	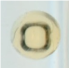	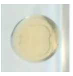	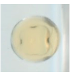	+<br>3/3
66	Saline	23.3	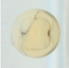	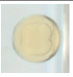	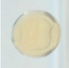	+<br>3/3
67	Saline	36.5	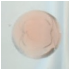	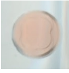	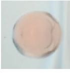	−<br>0/3

**Controls**				
**Sample ID**	**Medium**	**Concentration**	**OIL-TAS Image**	**OIL-TAS result**
PC3	PBS	10^3 copies/μL	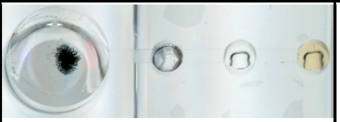	+
PC2	PBS	10^2 copies/μL	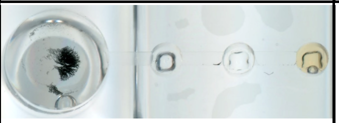	+
PC1	PBS	10^1 copies/μL	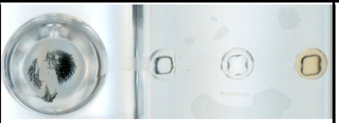	+
NC	PBS	0 copies/μL	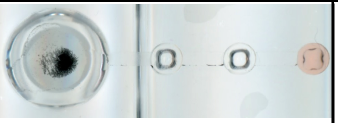	−

Supplementary Table 2: LAMP primer sequences

Primer Set	Sequence
***N* gene**	
F3	AACACAAGCTTTCGGCAG
B3	GAAATTTGGATCTTTGTCATCC
FIP	TGCGGCCAATGTTTGTAATCAGCCAAGGAAATTTTGGGGAC
BIP	CGCATTGGCATGGAAGTCACTTTGATGGCACCTGTGTAG
LF	TTCCTTGTCTGATTAGTTC
LB	ACCTTCGGGAACGTGGTT
**As1e**	
F3	CGGTGGACAAATTGTCAC
B3	CTTCTCTGGATTTAACACACTT
FIP	TCAGCACACAAAGCCAAAAATTTATTTTTCTGTGCAAAGGAAATTAAGGAG
BIP	TATTGGTGGAGCTAAACTTAAAGCCTTTTCTGTACAATCCCTTTGAGTG
LF	TTACAAGCTTAAAGAATGTCTGAACACT
LB	TTGAATTTAGGTGAAACATTTGTCACG
**RNase P**	
F3	TTGATGAGCTGGAGCCA
B3	CACCCTCAATGCAGAGTC
FIP	GTGTGACCCTGAAGACTCGGTTTTAGCCACTGACTCGGATC
BIP	CCTCCGTGATATGGCTCTTCGTTTTTTTCTTACATGGCTCTGGTC
LF	ATGTGGATGGCTGAGTTGTT
LB	CATGCTGAGTACTGGACCTC
