# Supplementary material for: Oil immersed lossless total analysis system for integrated RNA extraction and detection of SARS-CoV-2
Source: Nat Commun. 2021 Jul 14;12:4317. doi: 10.1038/s41467-021-24463-4 (PMC8280165; doi:10.1038/s41467-021-24463-4)
Supplement: Supplementary file 5 — Reporting Summary [file 41467_2021_24463_MOESM5_ESM.pdf]

nature research

Corresponding author(s): David J. Beebe

Last updated by author(s): Jun 9, 2021

Reporting Summary

Nature Research wishes to improve the reproducibility of the work that we publish. This form provides structure for consistency and transparency in reporting. For further information on Nature Research policies, see our Editorial Policies and the Editorial Policy Checklist.

Statistics

For all statistical analyses, confirm that the following items are present in the figure legend, table legend, main text, or Methods section.

n/a	Confirmed	
☐	☒	The exact sample size (*n*) for each experimental group/condition, given as a discrete number and unit of measurement
☐	☒	A statement on whether measurements were taken from distinct samples or whether the same sample was measured repeatedly
☐	☒	The statistical test(s) used AND whether they are one- or two-sided<br>*Only common tests should be described solely by name; describe more complex techniques in the Methods section.*
☒	☐	A description of all covariates tested
☒	☐	A description of any assumptions or corrections, such as tests of normality and adjustment for multiple comparisons
☐	☒	A full description of the statistical parameters including central tendency (e.g. means) or other basic estimates (e.g. regression coefficient) AND variation (e.g. standard deviation) or associated estimates of uncertainty (e.g. confidence intervals)
☒	☐	For null hypothesis testing, the test statistic (e.g. *F*, *t*, *r*) with confidence intervals, effect sizes, degrees of freedom and *P* value noted<br>*Give P values as exact values whenever suitable.*
☒	☐	For Bayesian analysis, information on the choice of priors and Markov chain Monte Carlo settings
☒	☐	For hierarchical and complex designs, identification of the appropriate level for tests and full reporting of outcomes
☒	☐	Estimates of effect sizes (e.g. Cohen's *d*, Pearson's *r*), indicating how they were calculated

Our web collection on statistics for biologists contains articles on many of the points above.

Software and code

Policy information about availability of computer code

Data collection	OIL-TAS images were acquired using Epson Scan (version 3.9.2.0US).
Data analysis	Data analysis was performed using Microsoft Excel (version 16.43), GraphPad Prism (version 6.01), and Roche LightCycler 480 software (version 1.5.1.62)

For manuscripts utilizing custom algorithms or software that are central to the research but not yet described in published literature, software must be made available to editors and reviewers. We strongly encourage code deposition in a community repository (e.g. GitHub). See the Nature Research guidelines for submitting code & software for further information.

Data

Policy information about availability of data

All manuscripts must include a data availability statement. This statement should provide the following information, where applicable:

- Accession codes, unique identifiers, or web links for publicly available datasets
- A list of figures that have associated raw data
- A description of any restrictions on data availability

The data supporting the findings of this study are available within the paper and its supplementary information files.

Field-specific reporting

Life sciences study design

All studies must disclose on these points even when the disclosure is negative.

Sample size	Sample size calculation was not performed. The sample size of 57 SARS-CoV-2 positive and 10 SARS-CoV-2 negative clinical NP swab samples was based on availability of residual clinical samples. For non-clinical samples, sensitivity and reproducibility of the assay was validated with 10-fold serial dilutions of heat-inactivated SARS-CoV-2 viral particles across 3 orders of magnitude with 2 to 10 replicates per concentration.
Data exclusions	No data was excluded from analysis.
Replication	OIL-TAS experiments were performed with at least 2 successful replicate runs.
Randomization	Clinical NP swab samples were randomly selected for testing using OIL-TAS. Sample randomization was not relevant for other experiments as samples are processed identically in parallel within each experiment, and each unit of the OIL-TAS device is of identical design.
Blinding	Clinical NP swab sample IDs were randomized for blinding before performing testing using OIL-TAS. For all other experiments, samples were not blinded as the investigators had to prepare serial dilutions of inactivated viral particle samples at known concentrations prior to each experiment.

Reporting for specific materials, systems and methods

We require information from authors about some types of materials, experimental systems and methods used in many studies. Here, indicate whether each material, system or method listed is relevant to your study. If you are not sure if a list item applies to your research, read the appropriate section before selecting a response.

Materials & experimental systems

n/a	Involved in the study
☒	☐ Antibodies
☐	☒ Eukaryotic cell lines
☒	☐ Palaeontology and archaeology
☒	☐ Animals and other organisms
☒	☐ Human research participants
☒	☐ Clinical data
☒	☐ Dual use research of concern

Methods

n/a	Involved in the study
☒	☐ ChIP-seq
☒	☐ Flow cytometry
☒	☐ MRI-based neuroimaging

Eukaryotic cell lines

Policy information about cell lines

Cell line source(s)	Human A549 lung cancer cells were sourced from ATCC.
Authentication	The cell line used (A549) was not authenticated for this study.
Mycoplasma contamination	The cell line used (A549) was not tested for mycoplasma contamination in this study.
Commonly misidentified lines (See ICLAC register)	Commonly misidentified cell lines were not used in this study
