# Supplementary material for: Oil immersed lossless total analysis system for integrated RNA extraction and detection of SARS-CoV-2
Source: Nat Commun. 2021 Jul 14;12:4317. doi: 10.1038/s41467-021-24463-4 (PMC8280165; doi:10.1038/s41467-021-24463-4)
Supplement: Supplementary file 6 — Description of Additional Supplementary Files [file 41467_2021_24463_MOESM6_ESM.pdf]

Title: Supplementary Movie 1:
Description: Sample extraction using OIL-TAS. Magnetic beads were magnetically transported from the sample well through 2 wash wells and into the detection well by dragging the OIL-TAS device from right to left on a magnetic extractor. The wash and detection wells were added with droplets of food coloring for visualization.

Title: Supplementary Movie 2:
Description: Extraction channel height screening. Side view of an OIL-TAS device with varying extraction channel heights ranging from 100 µm to 600 µm. Magnetic beads were magnetically transported through the extraction channels and water droplets (stained yellow with food coloring for visualization).

Title: Supplementary Movie 3:
Description: OIL-TAS stability. An OIL-TAS device containing droplets of food coloring was mechanically agitated on a rocker at 30 RPM and on an orbital shaker at 900 RPM to demonstrate the stability of droplets within the device.
